# Purinoceptor P2K1/DORN1 Enhances Plant Resistance Against a Soilborne Fungal Pathogen, *Rhizoctonia solani*

**DOI:** 10.3389/fpls.2020.572920

**Published:** 2020-09-25

**Authors:** Sonika Kumar, Diwaker Tripathi, Patricia A. Okubara, Kiwamu Tanaka

**Affiliations:** ^1^Department of Plant Pathology, Washington State University, Pullman, WA, United States; ^2^Wheat Health, Genetics and Quality Research Unit, USDA-ARS, Pullman, WA, United States

**Keywords:** extracellular ATP, damage-associated molecular patterns, purinoceptor P2K1, root disease, *Rhizoctonia solani*

## Abstract

The purinoceptor P2K1/DORN1 recognizes extracellular ATP, a damage-associated molecular pattern (DAMP) released upon cellular disruption by wounding and necrosis, which in turn, boost plant innate immunity. P2K1 is known to confer plant resistance to foliar biotrophic, hemi-biotrophic, and necrotrophic pathogens. However, until now, no information was available on its function in defense against root pathogens. In this report, we describe the contribution of P2K1 to resistance in *Arabidopsis* against *Rhizoctonia solani*, a broad host range, necrotrophic soilborne fungal pathogen. In pot assays, the *Arabidopsis* P2K1 overexpression line *OxP2K1* showed longer root length and a greater rosette surface area than wild type in the presence of the pathogen. In contrast, the knockout mutant *dorn1-3* and the double mutant *rbohd/f*, defective in two subunits of the respiratory burst complex NADPH oxidase, exhibited significant reductions in shoot and root lengths and rosette surface area compared to wild type when the pathogen was present. Expression of *PR1*, *PDF1.2*, and *JAZ5* in the roots was reduced in *dorn1-3* and *rbohd/f* and elevated in *OxP2K1* relative to wild type, indicating that the salicylate and jasmonate defense signaling pathways functioned in resistance. These results indicated that a DAMP-mediated defense system confers basal resistance against an important root necrotrophic fungal pathogen.

## Introduction

The necrotrophic soilborne pathogen *Rhizoctonia solani* infects more than 250 plant species, including corn, potato, soybean, pulses, brassicas, and small-grain cereals. *Rhizoctonia solani* primarily infects the below-ground organs of the plant (Weinhold and Sinclair, 1996; Keijer et al., 1997), resulting in damping-off, root rot and bare patch of cereals, brown patch of turfgrass, black scurf of potato, and sheath blight of rice (Sneh et al., 1991; DeShields et al., 2018). For example, *R. solani* anastomosis group 8 (AG-8), causes chronic and acute yield losses from 10-30% in dryland cereal production systems of the Pacific Northwest, USA and up to 100% in parts of the world, amounting to billions of dollars of annual losses to agriculture worldwide (Okubara et al., 2014; Okubara et al., 2019). It is a broad host range pathogen, causing disease on a variety of crop plants, including canola and other brassicas. *Rhizoctonia solani* AG2-1 is the premier causal agent of Rhizoctonia damping-off of brassicas and pulses (Paulitz et al., 2006; Sturrock et al., 2015). It is frequently associated with wheat roots but generally is less pathogenic relative to *R. solani* AG-8 on both wheat and brassicas (Sturrock et al., 2015). Management of Rhizoctonia root diseases has been under development for decades. Genetic resistance to *R. solani* AG-8 has been reported for wheat (Okubara et al., 2009; Mahoney et al., 2016; Mahoney et al., 2017) but is not readily deployable in wheat breeding programs due to the multigenic nature of resistance and absence of molecular markers (Okubara et al., 2019). However, a broad-spectrum resistance gene from rice that confers *R. solani* resistance in *Arabidopsis* has been recently described (Maeda et al., 2019) and would be more readily deployable than multigenic resistance. Foliar plant defense activators such as probenazole are not effective for crop protection from *R. solani* infection (Kouzai et al., 2018). Crop rotation can have limited utility due to the exceptionally broad host range of *R. solani*. Biological control has not been practiced in large-scale production systems, but one study demonstrated that the use of hypovirulent *Rhizoctonia* isolates was effective in protecting against virulent isolates (Sharon et al., 2011). Current management practices include direct seed or minimum tillage, applications of nitrogen at the time of planting, and reduction of weeds and volunteers using herbicides (Mahoney et al., 2016).

Molecular genetic approaches have been successful in boosting plant defense to *R. solani* infections. For example, rice plants with increased jasmonate (JA)-mediated defense responses and ethylene (ET) production showed enhanced resistance to *R. solani* (Peng et al., 2012; Helliwell et al., 2013). Using *Arabidopsis*, researchers have demonstrated that the heterologous expression of a germin-like protein from sugar beet enhanced *R. solani* resistance (Knecht et al., 2010). Interestingly, NADPH oxidase components, such as RBOHD and RBOHF, were also suggested to play an important role in plant resistance to *R. solani* (Foley et al., 2013). Introduced defense genes provided about 50% protection against *Rhizoctonia* and *Pythium* in wheat (Okubara et al., 2014). Although the genes described above have provided plant resistance to *R. solani*, more genetic information is needed to effectively control this difficult disease.

Accumulating evidence suggests that extracellular ATP is released, triggering plant responses to various biotic and abiotic stresses (Dark et al., 2011; Ramachandran et al., 2019). Extracellular ATP is a damage-associated molecular pattern or DAMP (Tanaka et al., 2014). In response to cellular disruption by wounding, necrotizing invasion or predation, ATP is released into the apoplast and perceived by the plant cell surface purinoceptor P2K1 (also known as DORN1) (Choi et al., 2014). Upon ATP binding to the extracellular lectin domain of the purinoceptor (Nguyen et al., 2016), its intracellular kinase domain is activated (Chen et al., 2017), which subsequently results in the activation of a number of intracellular signaling pathways for the reprogramming of many plant defense-related genes (Jewell et al., 2019). Overexpression of P2K1 enhances plant resistance against various foliar pathogens, such as *Phytophthora brassicae* (biotrophic oomycete), *Pseudomonas syringae* (hemibiotrophic bacterium), and *Botrytis cinerea* (necrotrophic fungus*)* (Bouwmeester et al., 2011; Balagué et al., 2017; Chen et al., 2017; Tripathi et al., 2018), suggesting that extracellular ATP plays an important role in plant defense against a broad range of pathogens. However, there is no report on whether extracellular ATP enhances plant resistance to root necrotrophic pathogens such as *R. solani*.

We hypothesized that extracellular ATP acts as a DAMP to enhance root resistance to the necrotizing root pathogen *R. solani*. Here, we used the *Arabidopsis-R. solani* pathosystem to assess plant defense responses mediated by extracellular ATP signaling. The model plant *Arabidopsis* can be infected with two different *R. solani* anastomosis groups, AG2-1 and AG-8 (Foley et al., 2013). Using these fungal strains, we evaluated the susceptibility in *Arabidopsis* wild-type plants (WT), DAMP-related knockout mutants and plants overexpressing the P2K1 receptor. Our results demonstrated that P2K1 mediates a root defense response against both *R. solani* AG-8 and AG2-1. This is the first report of the role of extracellular ATP for resistance to the root pathogen *R. solani*.

## Materials and Methods

### Plant Materials

All *Arabidopsis* genotypes used in the studies were in the ecotype Col-0 background. The knockout mutant *dorn1-3*, the overexpression line *OxP2K1*, and *rbohd/f* (a double knockout mutant of two NADPH oxidase subunits) were previously reported (Torres et al., 2002; Choi et al., 2014). Seeds were surface sterilized and plated onto half-strength Murashige & Skoog (MS) medium containing 2.2 g/L MS salt with vitamins (Caisson Labs Inc., Smithfield, UT), 1% (w/v) sucrose, 1% (w/v) agar, and 0.05% (w/v) MES (pH 5.7) in a square petri dish. Seeds were kept in the dark at 4°C for 3 days to synchronize germination and transferred to a growth chamber (Conviron Inc., Winnipeg, Canada) at 22°C under a 16-h light/8-h dark cycle (100 µmol m^-2^ s^-1^ light intensity). The petri dishes were oriented vertically during plant growth.

### Preparation and Enumeration of *Rhizoctonia solani* AG-8 Inoculum

*Rhizoctonia solani* AG-8 isolate C1 was cultured on potato dextrose agar (PDA) for up to 5 days (Okubara et al., 2009). Substrate for the inoculum was prepared by autoclaving feed-quality oats for 60 min per day on two consecutive days, with a 20- to 24-h period of cooling in between (Okubara et al., 2009). Approximately 15–20 agar cubes (~5 mm^3^) from the leading edge of the fungal colony were distributed among the cooled oats by gentle shaking. Inoculated oats were incubated in the dark for 3 weeks at 23°C. The oats were shaken gently every seven days for 2 weeks to redistribute the fungi. In the third week, the inoculum was air dried in a laminar hood on clean Kraft paper for 24–48 h. Dried inoculated oats were homogenized using a coffee grinder and passed through 1,000 µm and 250 µm sieves (Okubara et al., 2009). The ground inoculum was stored at -20°C for up to 3 weeks. *Rhizoctonia* colonization was quantified immediately before use from triplicate suspensions of 100 mg inoculum in 5 ml of water and 1:10 dilutions of each suspension. Two-hundred microliters of each suspension were plated on water agar containing 100 µg mL^-1^ of chloramphenicol. Colonies were counted for 3 successive days under a dissecting scope. Colony forming units (CFU) per gram of oat was the average of the cumulative colony counts for each dilution.

### Comparison of *Rhizoctonia solani* AG-8 and AG2-1 Pathogenicity

Surface-sterilized seeds of *Arabidopsis* (WT of Col-0) were germinated for 11 days on 0.8% M9 agar and then transferred to 4” pots containing pasteurized Sunshine Potting Mix #4 moistened with Miracle Gro solution (0.9 g/L). Soil was infested immediately prior to sowing with 0 or 50 CFU g^-1^ soil of *R. solani* AG2-1 or AG-8 isolate C1. Plants were grown at 22°C in 16-h light/8-h dark in an environmental growth chamber, with Miracle Gro supplements. Seedling weight and root morphometric analysis (total root length) were obtained after 14 days of infection. Non-infested soil served as the control.

### *Rhizoctonia solani* AG-8 Infection of Soil-Grown Plants

Eleven-day-old *Arabidopsis* seedlings were used for fungal infection in pots. Seedlings were transferred to a soil/sand mixture with a 1:1 (v:v) ratio containing *R. solani* AG-8 C1 inoculum in ground oats at a final population density of 150 CFU g^-1^ soil. No-pathogen controls were included for each genotype. Plants were grown for 2 weeks in a growth chamber at 22°C with 70% humidity under a 12-h light/12-h dark cycle (150 µmol m^-2^ s^-1^ light intensity), with 50 mL water per pot (1.50” × 3.38” × 2.27”) added on alternate days. At the time of harvest, water was added to the pot to loosen the soil and minimize root breakage. Eight replicates of the control and pathogen-challenged samples were evaluated in each experiment. The experiment was repeated three times. To evaluate the fungal growth in roots, total DNA was extracted and subjected to PCR-based quantification using primers for the AG-8 ITS region: AG-8_F (AGTTGGTTGTAGCTGGTCCATTAAT) and AG-8_R (AGTAGACAGAGGGGTCCAATAAATGA) (Budge et al., 2009). The data were normalized to an *Arabidopsis* reference gene, *AtSAND* (At2g28390).

### Morphometric Analysis of Soil-Grown Plants

Rosette areas and total root length of all the plants were measured from digital images after 14 days of growth in infested soil. Rosette area was quantified *in situ* using ImageJ (https://imagej.nih.gov/ij/). The roots were washed to remove soil and debris. Individual root systems were stored at 4°C between wet paper towels in polythene bags for about 24 h. Roots were transferred to a glass tray (25 × 25 × 2 cm), and water was added to just submerge the roots. The roots were carefully spread to minimize tangles and crossovers. Digital images were acquired using the Epson Twain Pro scanner at 600 dpi resolution. Total root length (cm) was determined for each root system using WinRHIZO Regular software (ver. 2016b, Regent Instruments, Inc., Quebec, Canada).

### Total RNA Extraction and Real-Time PCR

Total RNA in roots was extracted using a Quick-RNA Microprep kit (Zymo Research, Irvine, CA). Frozen tissues were powdered using ceramic beads for 60 s in a Mini-bead beater (Biospec, Bartlesville, OK). One microgram of total RNA was used to synthesize the first strand of cDNA using the iScript cDNA synthesis kit (Bio-Rad, Hercules, CA). Real-time PCR quantification of *PR1*, *PDF1.2* and *JAZ5* transcripts was performed using the SsoAdvanced SYBR Green Supermix kit on the CFX96 Touch Real-Time System (Bio-Rad, Hercules, CA). Thermal cycling conditions were composed of initial denaturation for 3 min at 95°C, followed by 40 cycles of denaturation for 10 s at 95°C and annealing and extension for 30 s at 60°C. The melting protocol was 65 to 95°C (increments 0.5°C/5 s) to ensure a unique PCR product was produced. *AtUBQ10* (At4g05320) or *AtSAND* genes were used to normalize the expression data. The Cq values of three biological replicates were used to calculate the expression of genes using the 2^-(ΔΔCq) equation (Schmittgen and Livak, 2008).

### *Rhizoctonia solani* AG2-1 Infection of Young Seedlings in the Presence of ATP

*R. solani* AG2-1 was grown on PDA for 2–3 days. A single agar plug (5-mm diameter) with actively growing fungus was placed in the middle of an MS plate amended with 100 µM ATP (Sigma-Aldrich, St. Louis, MO). Control plates did not contain exogenously added ATP. Ten to twelve 10-day-old *Arabidopsis* seedlings were placed around the fungal plug with the root tips facing the plug. After incubation for 2 days, the seedlings were washed three times with 3% (v/v) H_2_O_2_ and sterile water and then subjected to PCR-based quantification using primers for the AG2-1 ITS region. Primer sequences used are as follows: Rs2.1/8F (GTTGTAGCTGGCCCATTCATTTG) and Rs2.1/8R (GAGCAGGTGTGAAGCTGCAAAAG) (Okubara et al., 2008). The data were normalized to an *Arabidopsis* reference gene, *AtSAND*. The experiment was repeated at least three times.

### Statistical Analysis

All experiments were performed independently at least three times or more. The results were shown as the mean ± SE. The significant differences among the means were analyzed using Student’s t-test or an ANOVA model and a *post hoc* analysis using a t-test (P-value < 0.05).

## Results

### Pathogenicity of *R. solani* AG-8 and AG2-1 on *Arabidopsis*

**Figure 1:**
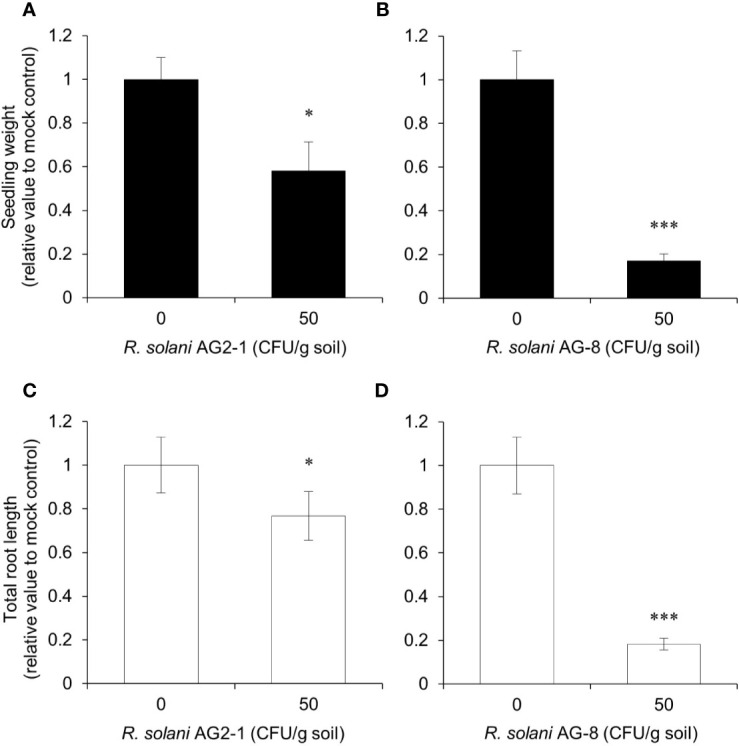
Pathogenicity assay for *Rhizoctonia solani* AG2-1 and AG-8 against *Arabidopsis* Col-0 WT. Eleven-day-old seedlings were planted in pasteurized soil infested with 0 and 50 CFU g^-1^ of *R. solani* AG2-1 **(A**, **C)** or AG-8 **(B, D)**. At 14 days post-infection, total seedling weight **(A, B)** and total root length **(C, D)** were quantified. Means ± S.E. are the average of 8 to 16 plants. Data are presented as the relative value to mock control (0 CFU g^-1^ soil). Asterisks indicate statistically significant differences compared with the control: Student’s t test, *P < 0.05 and ***P < 0.001.

First, a pathogenicity comparison was carried out to determine which AG caused more disease on *Arabidopsis* and would be more likely to distinguish the responses of the mutants. *Arabidopsis* Col-0 plants showed differential susceptibility to *R. solani* AG2-1 compared to *R. solani* AG-8 in the pathogenicity assays (**Figure 1**). Reductions in seedling weight and total root length were less than twofold in the soil infested with 50 CFU g^-1^ to *R. solani* AG2-1 but approached fivefold for *R. solani* AG-8 relative to the non-infested control. The findings supported observations that the latter pathogen produced more severe disease symptoms than the former for *Arabidopsis* Col-0 plants. Therefore, subsequent experiments were focused on *R. solani* AG-8.

### P2K1 Confers Plant Resistance Against *R. solani*

**Figure 2:**
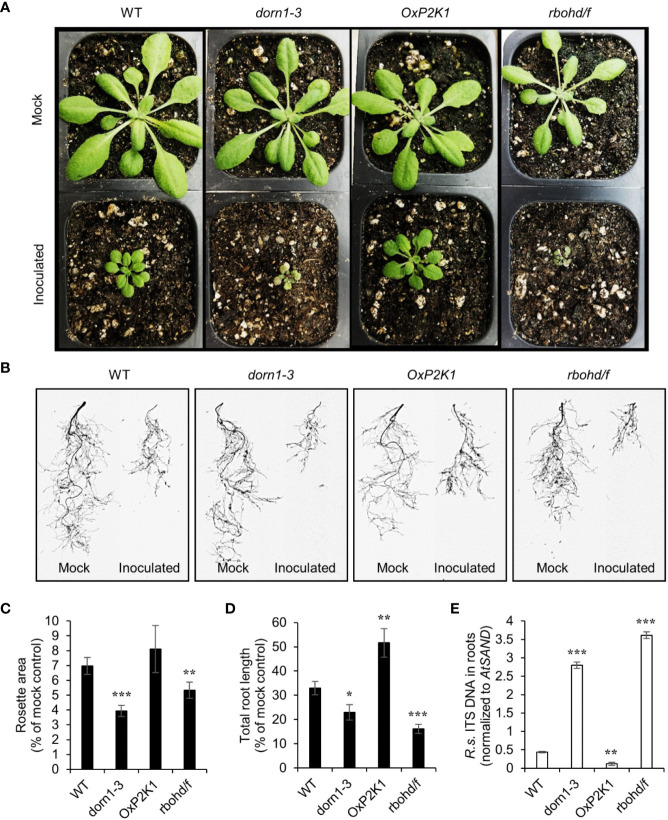
Effect of *Rhizoctonia solani* AG-8 infection on the aboveground rosette growth and the root growth of *Arabidopsis*. Eleven-day-old *Arabidopsis* seedlings were transferred to noninfested soil (mock) or soil infested with *R. solani* AG-8 C1 (150 CFU/g soil). Pictures of aboveground **(A)** and scan images of the root systems **(B)** were taken at 14 days of growth in the soil. The rosette areas of all four genotypes were measured as the smallest circle area enclosed by the convex hull of the rosette **(C)**. The total root length was quantified using the WinRHIZO **(D)**. Data are presented as the mean percentage ± S.E. (n = 8) relative to mock control. The level of infection in the roots quantified by real-time PCR based **(E)**. Abundance is expressed as the ratios of the fungal-specific ITS region relative to the *Arabidopsis* reference gene *AtSAND*. The values are the means ± S.E. of three biological replicates. Asterisks indicate statistically significant differences compared with the WT control: Student’s t test; *P < 0.05; **0.001 < P < 0.01; ***P < 0.001.

To measure the contribution of the purinoceptor P2K1 to plant resistance to the soilborne pathogen *R. solani* AG-8, we performed a pot-based fungal pathogenicity assay using four genotypes of *Arabidopsis* grown in soil infested with the pathogen. The NADPH oxidase double mutant *rbohd/f* was used as a positive control since it was reported to show complete loss of resistance to *R. solani* (Foley et al., 2013). After only one week of growth in soil containing 150 CFU g^-1^ of pathogen, severe stunting of aerial growth was observed. Reduction in rosette area was an indicator of underground-derived stress, usually due to root loss, in plants infected with soilborne pathogens (Mahoney et al., 2016; Julkowska et al., 2016). **Figure 2A** shows the rosette leaves of all four genotypes grown in noninfested and infested soils for 14 days. Both *dorn1-3* and *rbohd/f* showed a very high reduction in total rosette area (**Figure 2C** and **Supplementary Figure S1A**). Based on a visual observation, *dorn1-3* and *rbohd/f* also showed a yellow to tan chlorosis on their leaves (**Figure 2A**). In contrast, *OxP2K1* showed the least reduction compared to wild type (WT) plants although the difference between them are not statistically significant (**Figure 2C**). Similar results were obtained with infested field soil locally collected from the Spillman Agronomy Farm, Pullman, WA (**Supplementary Figures S2A, C**).

Total root length is an informative phenotype for assessing plant resistance against soilborne pathogens (Okubara et al., 2009). After 14 days of growth in infested soil, significant reduction (>50%) in total root length was observed in the infected plants compared to the noninfected control plants (**Figure 2B**). Similar to the data in the rosette area, the total root length values in both *dorn1-3* and *rbohd/f* were severely reduced by fungal infection. The *OxP2K1* and WT lines exhibited lesser degrees of reduction (**Figure 2D** and **Supplementary Figure S1B**). The *OxP2K1* line sustained the least reduction compared to all other genotypes. We also measured the infection level of *R. solani* in the root tissues by real-time PCR (**Figure 2E**), showing a similar trend as seen in the fungal biomass result in the infected roots. Similar results were obtained when field soil was infested (**Supplementary Figures S2B, D**). Altogether, our observations showed that *dorn1-3* and *rbohd/f* were highly susceptible, while *OxP2K1* was more resistant than the WT. The data suggested that P2K1 is involved in a basal plant resistance to *Rhizoctonia* infection, and that defects in DAMP signaling through P2K1 and reactive oxygen species (ROS) generation by the NADPH oxidases resulted in enhanced susceptibility.

### P2K1 Is Involved in the Upregulation of Defense-Related Genes During *R. solani* Infection

**Figure 3:**
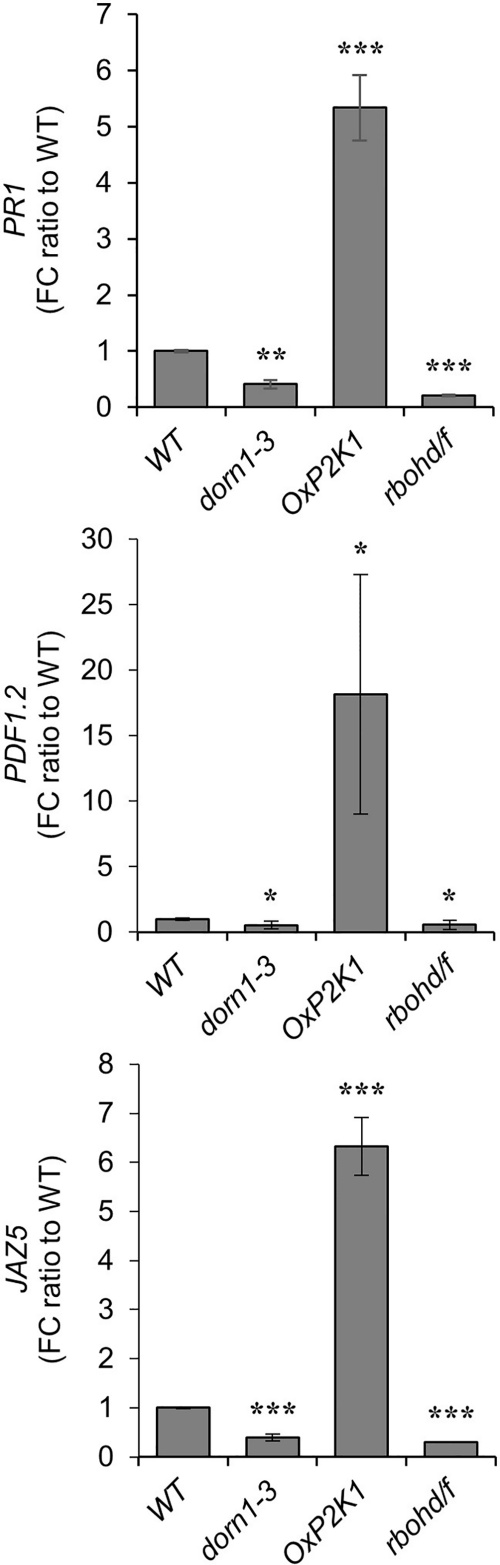
Expression of defense-related genes in the *Rhizoctonia solani* AG-8-infected roots. The expression of defense-related genes, *PR1*, *PDF1.2*, and *JAZ5*, were measured in the root tissues of the WT, *dorn1-3*, *OxP2K1*, and *rbohd/f* plants after growth for 14 days in noninfested and infested soils. Data are presented as the mean value of fold change (FC) ± S.E. (n = 5) relative to that of WT. Asterisks indicate statistically significant differences compared with the WT control: Student’s t test; *P < 0.05; **0.001 < P < 0.01; ***P < 0.001.

The expression of a salicylic acid (SA)-induced gene, pathogenesis-related *PR1* (Wildermuth et al., 2001), and two JA/ET-induced genes, the defensin *PDF1.2* and the JA-ZIM-domain protein *JAZ5* (Penninckx et al., 1998; Smirnova et al., 2017), were measured in the root tissues of the WT, *dorn1-3*, *OxP2K1*, and *rbohd/f* plants after growth for 14 days in noninfested and infested soil conditions. Consistent with a previous report (Foley et al., 2013), all these defense-related genes were upregulated after fungal infection in all genotypes tested, but highly upregulated in *OxP2K1* compared to the WT, and weakly upregulated in *dorn1-3 and rbohd/f* after fungal infection (**Figure 3**). Additionally, the expression level of the defense-related genes were comparable in mock-inoculated roots among all genotypes tested (**Supplementary Figure S3**). The result further demonstrated that the expression of the defense-related genes was dependent on the P2K1-mediated pathway only upon host damage. In other words, P2K1 plays an important role for the induction of defense-related genes during *R. solani* infection.

### Application of ATP Reduces Infectionof *R. solani* and Alleviates Stunting of Root Growth

**Figure 4:**
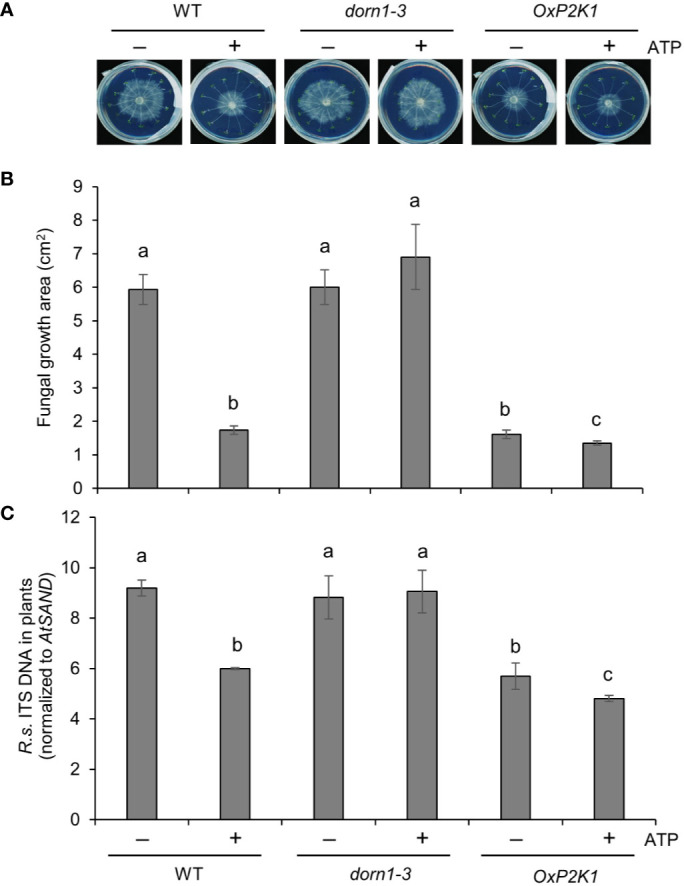
Extracellular ATP reduces *Rhizoctonia solani* AG2-1 infection in roots. **(A)** Representative pictures of *R. solani* infection in MS plates with wild type (WT), *dorn1-3*, and *OxP2K1* seedlings at 2 days post infection. **(B)** The area of fungal growth on the MS plates in **(A)**. **(C)** The level of infection in the seedlings quantified by real-time PCR. Abundance is expressed as the ratios of the fungal-specific ITS region relative to the *Arabidopsis* reference gene *AtSAND*. The values are the means ± SE of three biological replicates. Different letters indicate significant differences at P < 0.05.

It was important to do one experiment with *R. solani* AG2-1, the causal agent of the main *Rhizoctonia* disease, post-emergence damping off in the brassicas. This AG group would be useful to see the effect of exogenously applied ATP on fungal colonization (biomass) at a high resolution, even in the susceptible mutant lines. To this end, colonization of roots by *R. solani* AG2-1 was tested in a petri plate assay in the presence or absence of ATP. Fungal growth was visible 2–3 days after inoculation (**Figure 4**), and the infection level was estimated by measuring the fungal growth on the plates and also by quantifying the fungus inside or closely associated with the root tissue. The root-associated fungal biomass varied according to the genotype and ATP treatment. **Figure 4** shows that *R. solani* biomass was reduced in the *OxP2K1* line, although it was no difference in *dorn1-3*, in comparison to that for WT (**Figures 4A, B**). Additionally, exogenous ATP significantly reduced fungal biomass in roots of the WT and *OxP2K1* lines, whereas it failed to attenuate the fungal biomass in the *dorn1-3* mutant (**Figure 4B**). We also measured the infection level of *R. solani* in the root tissues by real-time PCR (**Figure 4C**), showing a similar trend as seen in the fungal biomass result on the plates. These findings suggest that extracellular ATP mediates defense against *R. solani* through P2K1.

## Discussion

The soilborne fungus *R. solani* is an economically important pathogen (Sneh et al., 1996) causing diseases primarily in the roots or tubers and secondarily in the stems of plants (Keijer et al., 1997; Weinhold and Sinclair, 1996). Although *R. solani* AG2-1 is typically associated with disease in the brassicas and pulses, it primarily causes post-emergence damping off, and isolates can vary in pathogenicity. In contrast, *R. solani* AG-8 causes more severe disease symptoms in the field, including pre-emergence damping off and bare patch (Khangura et al., 1999; T. Paulitz, personal communication). Our findings indicated that it was also the stronger pathogen of *Arabidopsis* in our pathogenicity assay and therefore preferable for generating disease in the *Arabidopsis* mutants under controlled conditions. *Arabidopsis* was the host of choice, given that mutants in eATP signaling were not available for other crops.

*Rhizoctonia solani* is a necrotroph that typically causes cellular damage to acquire nutrients from the host. Necrosis-mediated cellular disruption is a direct mechanism for releasing intracellular ATP into the extracellular matrix (i.e., apoplast). In animals, ATP is released as a DAMP signal when endogenous physiological factors and foreign entities cause cell damage and death, e.g., apoptosis, pyroptosis, and necrosis (Dosch et al., 2018). Similarly, in plants, a high concentration of cytoplasmic ATP is released into the extracellular spaces following exposure to abiotic and biotic stresses (Dark et al., 2011; Ramachandran et al., 2019), suggesting a role of extracellular ATP in stress responses.

In the present study, we hypothesized that extracellular ATP, released upon *R. solani*-induced cellular damage (i.e., necrosis), activates the purinoceptor P2K1, which initiates a signaling cascade leading to the induction of defense genes. Our data showed that the purinoceptor P2K1 mediated defense against a necrotrophic root infection. The overexpression line of P2K1 was more resistant to both anastomosis groups (AG-8 and AG2-1) of *R. solani* than the WT. The knockout mutant *dorn1-3* exhibited a high susceptibility to AG-8, but not to AG2-1 (this is probably due to different experiment systems used). In addition, ATP addition attenuated the fungal growth in roots of the WT and overexpression lines. This ATP-induced inhibition of fungal growth was abolished in the knockout mutant. These results demonstrated that extracellular ATP is involved in plant immune response to *R. solani* in roots, where the P2K1 receptor play an essential role to mediate ATP-induced defense. Given that there is no significant difference in the P2K1 expression upon *R. solani* inoculation (GSE26206) and ATP addition (GSE52610) based on previous transcriptomics (Foley et al., 2013; Choi et al., 2014; Jewell et al., 2019), the numbers of the purinoceptor is likely maintained same no matter when the plant cells get insulted by the fungal pathogen and exposed by extracellular ATP. This evidence further supports our hypothesis that extracellular ATP released upon fungal infection, not upregulation of the receptor, is critical for induction of defense responses to R. solani in the plant roots. To our knowledge, this study is the first demonstration of the role of extracellular ATP for basal resistance against a root pathogen, i.e., *R. solani*.

Reactive oxygen species produced by NADPH oxidases play a crucial role in resistance to several pathogens, such as *Alternaria brassicicola, Magnaporthe oryzae*, *P. parasitica*, *B. cinerea, and R*. *solani* (Segmueller et al., 2008; Pogany et al., 2009; Larroque et al., 2013; Foley et al., 2013; Nozaki et al., 2013; Li et al., 2015). Interestingly, our data with the knockout mutant of P2K1 showed a complete loss of resistance that was comparable to the ROS-deficient double mutant *rbohd/f*, suggesting that ROS generation was as important as extracellular ATP perception in the root defense response. Given that P2K1 directly interacts with and activates RBOHD by phosphorylation (Chen et al., 2017), it is possible that extracellular ATP released upon *R. solani* infection activates P2K1-RBOHD-mediated ROS signaling, thereby enhancing plant resistance to fungal infection.

Defense against *R. solani* has been linked to the overall effect produced by the signaling pathways of SA, ET, JA, abscisic acid, and auxin (Foley et al., 2013). In general, the plant defense response is a coordinated mechanism facilitated by the major defense hormones SA and JA/ET (Glazebrook, 2005; Spoel et al., 2007; Hillmer et al., 2017). Salicylic acid is a key modulator of local and systemic resistance. This molecule accumulates in tissues along with a coordinated expression of defense genes, including *PR* genes (Malamy et al., 1990; Métraux et al., 1990; Maleck et al., 2000). Jasmonic acid and ET are induced during necrotrophic pathogen infection, which induces the expression of several other defense genes, including defensins and proteinase inhibitors (Reymond and Farmer, 1998; Feys and Parker, 2000; Schenk et al., 2000; Devadas et al., 2002). A previous study showed that *R. solani* induces multiple defense hormone-related genes in potato sprouts (Lehtonen et al., 2008) and *Arabidopsis* (Foley et al., 2013). In this study, we confirmed that SA- and JA/ET-regulated genes were proportionally higher in *OxP2K1* and lower in the *dorn1* mutant during fungal infection, suggesting that the expression of these defense-related genes was dependent on the P2K1-mediated pathway upon host damage. Indeed, extracellular ATP induces various defense responses in parallel with plant defense hormones and through an independent mechanism (Jewell et al., 2019). Some of these responses are involved in enhancing resistance against the necrotroph *B. cinerea* mediated by extracellular ATP-JA-mediated synergistic signaling that required ROS as well as other second messengers, nitrous oxide, and calcium (Tripathi et al., 2018; Tripathi and Tanaka, 2018). Particularly, ROS and calcium signalings mutually interplay in plant immune response (Marcec et al., 2019). Additionally, the expression of many defense-related genes was further exaggerated in *OxP2K1*, in which ET-regulated genes were shown to be involved additional defense-related functions (Jewell and Tanaka, 2019).

Plants have basal resistance to necrotrophic pathogens, for which extracellular ATP reinforces plant defense responses *via* defense hormone pathways that may involve secondary metabolites, including tryptophan-derived compounds such as camalexin and glucosinolates, based on previous transcriptomic data (Jewell et al., 2019). To date, many efforts have been made to identify the resistance mechanism against *R. solani* in different crops. Genetic studies in wheat indicate that both single-gene and multigenic resistance are involved and that several mechanisms of resistance are likely, but until now, the molecular basis for basal resistance has been elusive. A better understanding of extracellular ATP signaling would contribute to breeding crop varieties with increased resistance to soilborne necrotrophic pathogens. Further studies are required to understand the complete mechanism by which extracellular ATP enhances defense against this and other soilborne pathogens, which might provide leads to new management strategies for growers. Very recently, putative orthologs of P2K1/DORN1 have been reported in banana, camelina, and wheat (Li et al., 2016; Okubara et al., 2019; Shan et al., 2020). Such functional studies using crops other than *Arabidopsis* would allow us to directly use the resistance mechanism against the necrotrophic pathogens.

## Data Availability Statement

All datasets presented in this study are included in the article/**Supplementary Material**.

## Author Contributions

PO and KT conceived and designed the study. SK, DT, PO, and KT performed research, analyzed data, and wrote the article.

## Funding

This work was supported by the National Science Foundation (grant no. IOS-1557813) and USDA NIFA (Hatch project no. 1015621) to KT and CRIS project number 2090-22000-017-00D to PO.

## Conflict of Interest

The authors declare that the research was conducted in the absence of any commercial or financial relationships that could be construed as a potential conflict of interest.
