# Supplementary material for: Purinoceptor P2K1/DORN1 Enhances Plant Resistance Against a Soilborne Fungal Pathogen, Rhizoctonia solani
Source: Front Plant Sci. 2020 Sep 25;11:572920. doi: 10.3389/fpls.2020.572920 (PMC7545828; doi:10.3389/fpls.2020.572920)
Supplement: Supplementary file 1 [file DataSheet_1.pdf]

Supplementary Materials

Purinoceptor P2K1/DORN1 enhances plant resistance against a soilborne fungal pathogen, *Rhizoctonia solani*

Sonika Kumar[1,2,a,c], Diwaker Tripathi[1,b,c], Patricia A. Okubara[2], and Kiwamu Tanaka[1,*]

[1] Department of Plant Pathology, Washington State University, Pullman, WA 99164, USA
[2] USDA-ARS, Wheat Health, Genetics and Quality Research Unit, Pullman, WA, 99164, USA
[a] Current address: Department of Plant and Environmental Sciences, Clemson University, Clemson, SC, 29634, USA
[b] Current address: Department of Biology, University of Washington, Seattle, WA 98195, USA
[c] Equal contribution to this work
* For correspondence: kiwamu.tanaka@wsu.edu

This file contains:

Supplementary Figure S1. Effect of *Rhizoctonia solani* infection on Arabidopsis growth.

Supplementary Figure S2. Effect of *Rhizoctonia solani* infection on the Arabidopsis growth in the field soil.

Supplementary Figure S3. Expression of defense-related genes in mock-inoculated roots.

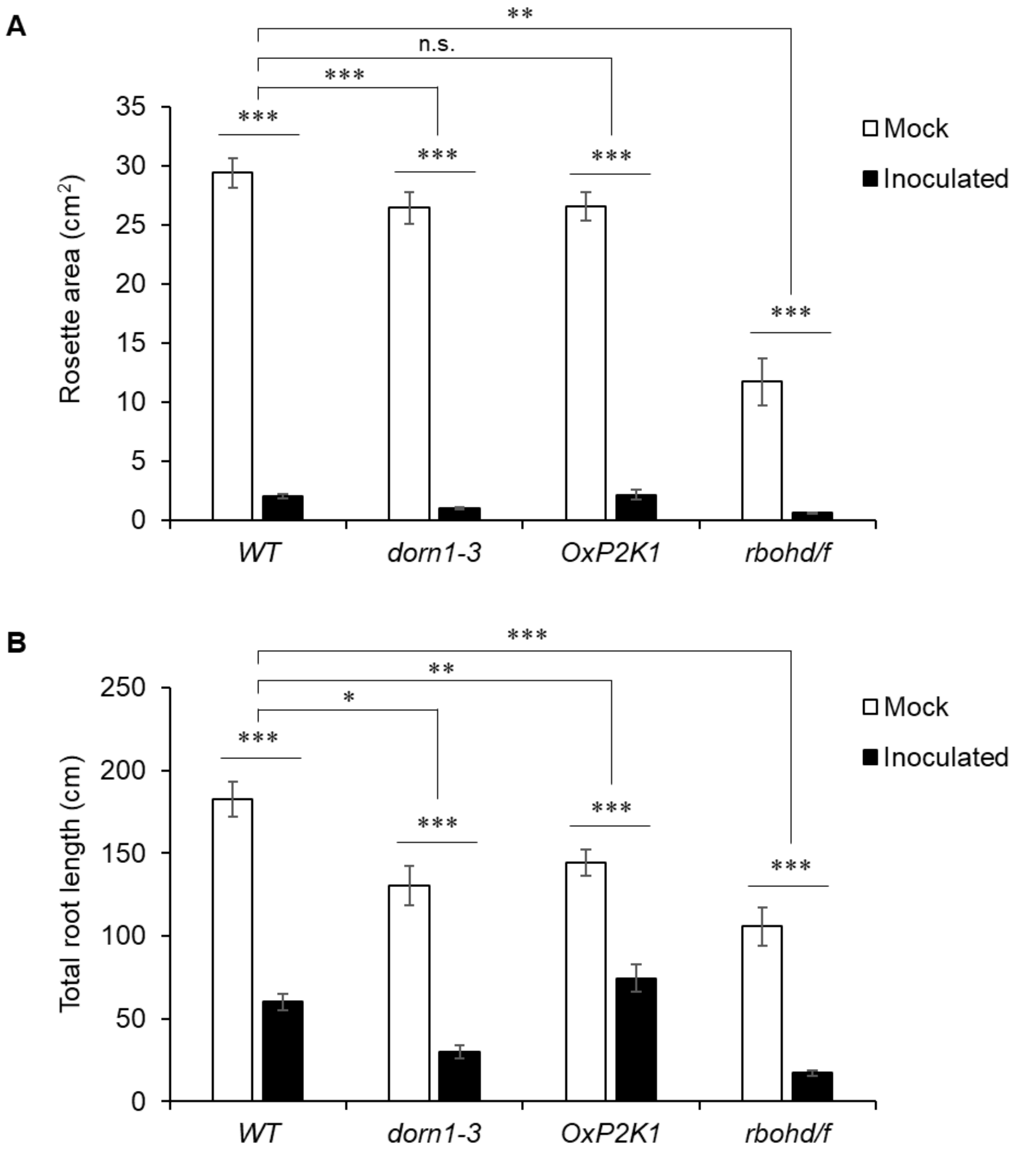

Supplementary Figure S1. Effect of *Rhizoctonia solani* infection on Arabidopsis growth.
(A) Rosette area and (B) total root length for mock (non-inoculated) and inoculated (150 CFU/g soil) plants. Data represent actual measured values of those shown Figure 2C and Figure 2D. Asterisks indicate statistically significant differences between mock vs. inoculated group or differences in relative values with respect to the WT control: * $P < 0.05$; ** $0.001 < P < 0.01$; *** $P < 0.001$; n.s. = not significant.

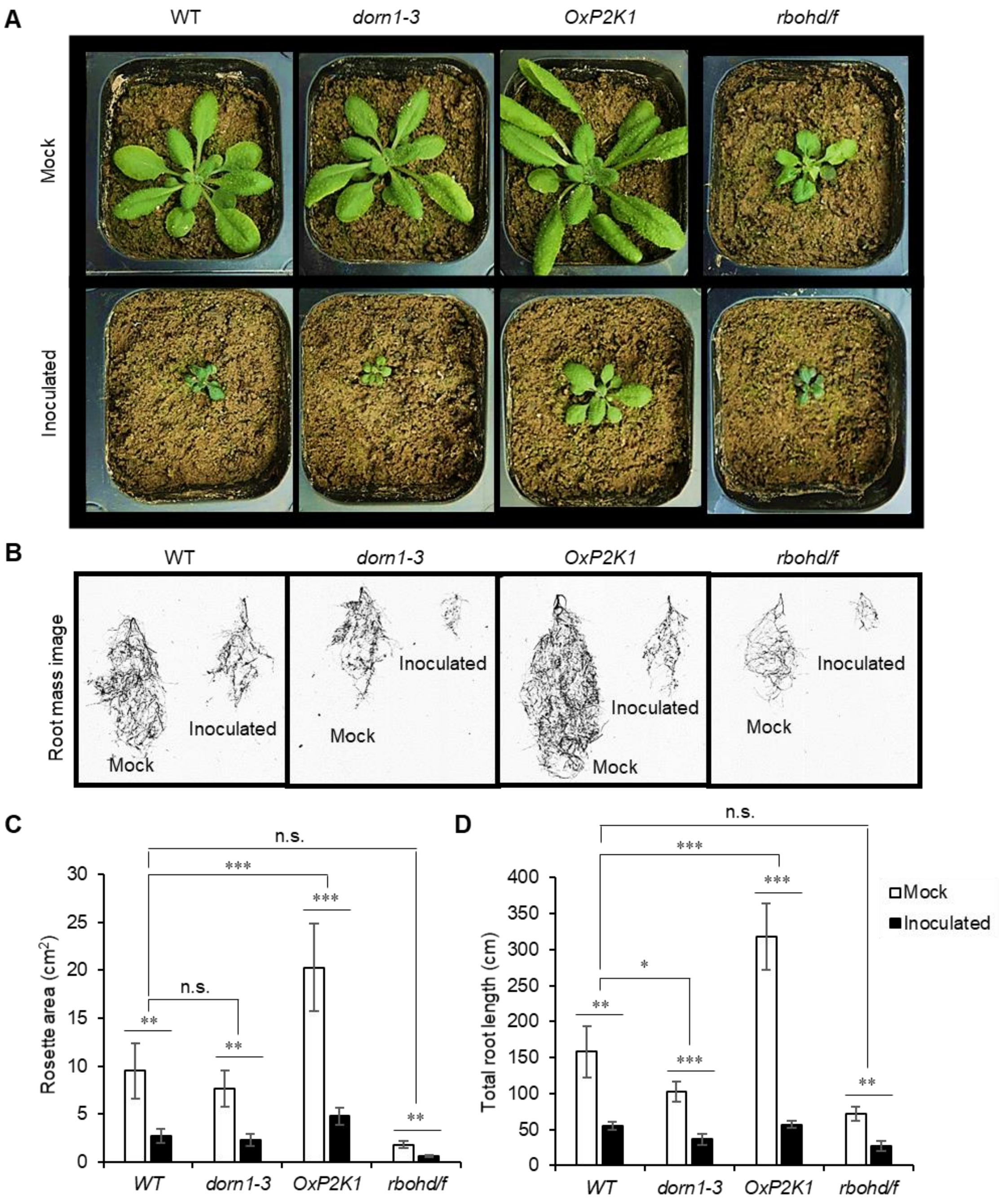

Supplementary Figure S2. Effect of *Rhizoctonia solani* infection on the Arabidopsis growth in the field soil. Eleven-day-old Arabidopsis seedlings were transferred to non-infested soil (mock) or soil infested with *R. solani* AG-8 C1 (100 CFU/g soil). The field soil was locally collected and autoclaved immediately before use. To eradicate other microbe contamination, e.g., oomycetes, the soil was soaked in metalaxyl solution (75 mg/L) before use. Pictures of aboveground (A) and scan images of the root systems (B) were taken at 14 days of growth in the soil. The rosette areas of all four genotypes were measured as the smallest circle area enclosed by the convex hull of the rosette (C). The total root length was quantified using the WinRHIZO (D). Data are presented as the mean value ± S.E. (n = 8). Asterisks indicate statistically significant differences between mock vs. inoculated group or differences in relative values with respect to the WT control: * P < 0.05; ** 0.001 < P < 0.01; *** P < 0.001; n.s. = not significant.

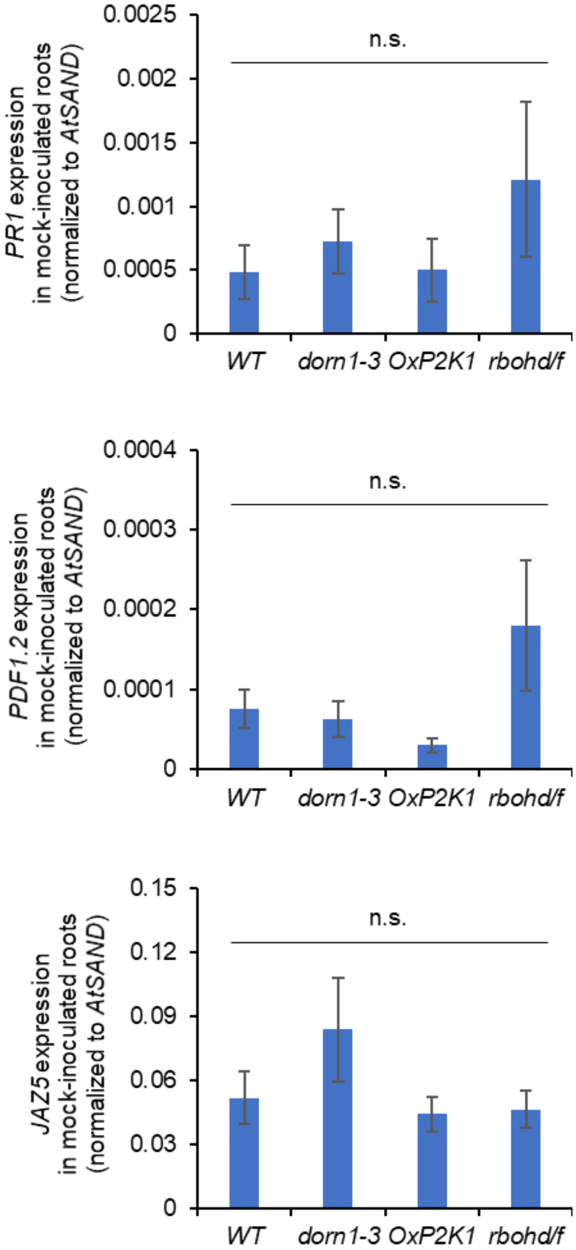

Supplementary Figure S3. Expression of defense-related genes in mock-inoculated roots. The expression of defense-related genes, *PR1*, *PDF1.2*, and *JAZ5*, was measured in the root tissues of the WT, *dorn1-3*, *OxP2K1*, and *rbohd/f* plants after growth for 14 days in noninfested soils. Data are presented as the mean value ± S.E. (n = 5) relative to that of the reference gene *AtSAND*. Two-way ANOVA showed no significant differences among groups (n.s.: P>0.05).
